# Profiling of Small Noncoding RNAs During Bovine Conceptus Elongation Identifies Let-7 as a Candidate Regulator of Proliferation and Differentiation

**DOI:** 10.3390/ani16081181

**Published:** 2026-04-13

**Authors:** Gabriela L. Murphy, Anna K. Goldkamp, Maria J. A. Lopes, Nicolle F. F. Bönmann, Matthew C. Lucy, Darren E. Hagen, João G. N. Moraes

**Affiliations:** 1 Department of Animal and Food Sciences, Oklahoma State University, Stillwater, OK 74078, USA; gablamb@okstate.edu (G.L.M.); maarauj@okstate.edu (M.J.A.L.);; 2 Division of Animal Sciences, University of Missouri, Columbia, MO 65211, USA

**Keywords:** let-7, miRNA, tRF, conceptus elongation

## Abstract

Small noncoding RNAs play critical regulatory roles in development across organisms. This study profiled microRNAs (miRNAs) and tRNA-derived fragments (tRFs) during bovine conceptus elongation. Elongating conceptuses were obtained via superovulation of eight Angus heifers. Twenty samples from ovoid (OV, *n =* 6; 0.5–3 mm), tubular (TUB, *n* = 7; 5–15 mm), and filamentous (FIL, *n* = 7; 20–34 mm) stages underwent small RNA sequencing. Differential expression of miRNAs and tRFs was analyzed using DESeq2, accounting for donor-sire effects. No tRFs showed differential abundance across any pairwise comparisons. For miRNAs, the expressions of six miRNAs were upregulated in OV versus TUB conceptuses (padj < 0.05), including four let-7 family members (bta-let-7g, bta-let-7f, bta-let-7a-5p, and bta-let-7c) and two additional miRNAs (bta-miR-224 and bta-miR-449a). Furthermore, there were 3 miRNAs differently abundant between the ovoid and filamentous transition (padj < 0.04), including two members of the let7 family (bta-let-7g and bta-let-7f) and bta-miR-449a. Predicted targets of these differentially abundant miRNAs were identified using miRanda. Enrichment analyses of the targeted genes included pathways regulating cellular proliferation, pathways in cancer, and immune-related pathways. The let-7 family, along with miR-449a and miR-224, are candidate regulators of the balance between cellular proliferation and differentiation during elongation, based on their differential abundance and in silico target predictions.

## Simple Summary

Early loss of pregnancy is a major cause of reduced reproductive efficiency in cattle and results in substantial economic losses for beef and dairy producers. Most pregnancy losses occur within the first month of gestation, which includes a critical period known as conceptus elongation, when the embryo rapidly grows and signals its presence to the mother to maintain pregnancy. Understanding the biological mechanisms that regulate this process is essential for developing strategies to improve pregnancy success. This study examined the role of small noncoding RNAs, molecules that help control gene activity, during conceptus elongation. Two types of these molecules, microRNAs and tRNA-derived fragments, were analyzed in conceptuses from Angus heifers at different developmental stages. Conceptuses were grouped as ovoid, tubular, or filamentous based on size and shape. The results showed that both the dam and sire influenced the levels of these regulatory molecules. While tRNA-derived fragments did not differ across developmental stages, several microRNAs changed significantly as the conceptus elongated. Many belonged to the let-7 family, along with miR-449a and miR-224, which are candidates for potentially regulating the balance between cellular proliferation and differentiation, required for successful elongation and pregnancy establishment.

## 1. Introduction

The eukaryotic genome comprises both coding and noncoding DNA sequences. Although some noncoding regions are transcribed, they do not translate into proteins but instead play important roles in regulating the expression of protein-coding genes [1]. Among the diverse classes of regulatory noncoding RNAs, microRNAs (miRNAs) and transfer RNA-derived fragments (tRFs) are characterized by short, single-stranded nucleotide sequences capable of influencing gene expression, primarily through post-transcriptional regulation [2,3].

**Figure 1:**
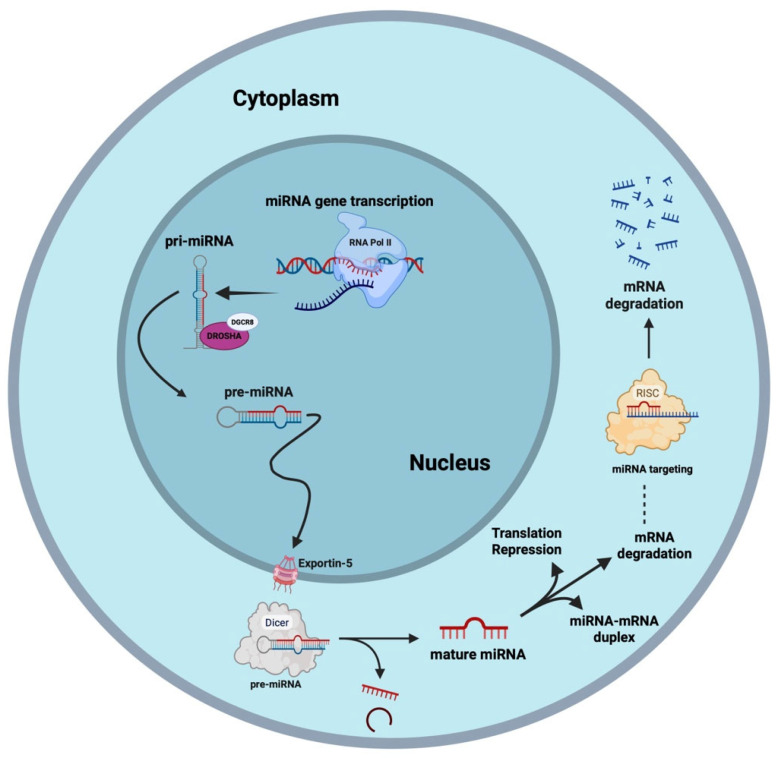
microRNA biogenesis and function. Canonical miRNA biogenesis initiates with transcription by RNA polymerase II/III, forming primary-miRNAs (pri-miRNA), long stem-loop structures. Still within the nucleus, pri-miRNAs are recognized and cleaved by the microprocessor complex (consisting of Drosha and DGCR8) to form the precursor-miRNAs (pre-miRNA), which are exported to the cytoplasm by the Exportin-5 complex. When in the cytoplasm, pre-miRNA is processed by Dicer, removing the terminal loop and resulting in a mature miRNA duplex. Both strands (5′ or 3′) derived from the mature mRNA can be loaded into Argonaute (AGO) proteins to form the miRNA-induced silencing complex (RISC). This multiprotein complex (RISC) uses the single-stranded miRNA as a template to recognize complementary mRNA for degradation. Created in BioRender https://BioRender.com/000x89c (accessed on 30 March 2026).

MicroRNAs are approximately 19 to 22 nucleotides (nt) in length, and function by binding to the Argonaute (AGO) protein to form the miRNA-induced silencing complex (miRISC). This complex decreases the expression of targeted transcripts, generally by binding to the 3′ untranslated region (UTR) of target mRNAs and inhibiting translation, promoting deadenylation, or inducing transcript degradation (Figure 1) [2,4].

**Figure 2:**
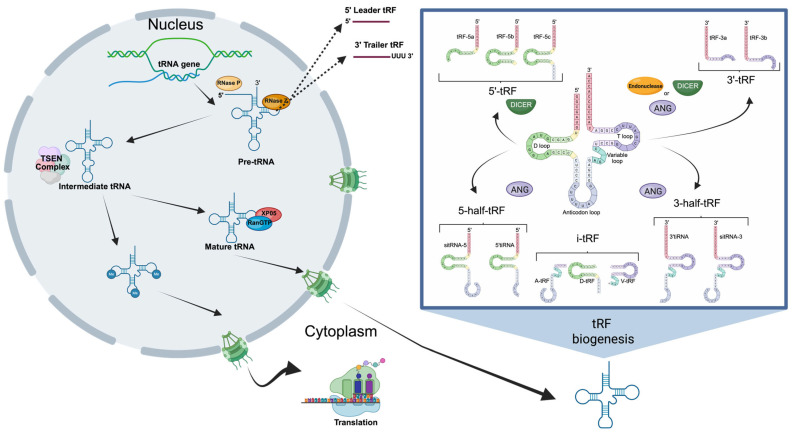
Transfer RNA (tRNA)-derived fragments biogenesis. During biogenesis, tRNAs are transcribed by RNA polymerase III into precursor tRNAs (pre-tRNA). Then pre-tRNA are converted into mature tRNA, a process involving the removal of the 5′ leader sequence by RNase P and removal of the 3′ trailer sequence by RNase Z. Mature tRNAs can be cleaved through either Dicer-dependent or Dicer-independent mechanisms, generating distinct classes of tRFs according to the cleavage site within the tRNA molecule: 5′ tRFs, 3′ tRFs, internal tRFs (i-tRF), and 5′ and 3′ halves. The tRNA halves are generated by ANG cleaving the middle of the anticodon loop, forming 5′ or 3′ halves. The 5′-tRF are derived from mature tRNAs after cleavage at the D-loop by Dicer. Furthermore, 3′-tRFs are derived from mature tRNAs after cleavage at the T-loop by distinct endonucleases. Lastly, the i-tRF originates from the internal region of the mature tRNAs, and may contain the tRNA anticodon loop, and D- and T-loops. Created in BioRender https://BioRender.com/ksltuc1 (accessed on 30 March 2026).

tRNA-derived fragments, as the name implies, are generated from transfer RNAs (tRNAs), a class of noncoding RNA with a well-established role in translation, serving as adaptor molecules that deliver amino acids to the growing polypeptide chain [5]. Traditionally, tRNAs were believed to have a role restricted to translation. Emerging evidence indicates that tRNAs can also influence gene expression through various mechanisms, including codon usage bias [6], post-transcriptional modifications, aminoacylation levels, and tRFs [7]. Mature or precursor tRNAs can be selectively cleaved to generate tRFs [8], which are approximately 14–30 nucleotides in length with regulatory roles implicated in various biological processes [9,10]. These fragments can function in a miRNA-like manner, inhibiting translation through mRNA competition, displacement of initiation factors, or by promoting stress granule formation (Figure 2). Based on their cleavage site, biogenesis, and relative length, tRFs can be grouped into five classes: 5′ tRFs, 3′ tRFs, internal tRFs (i-tRFs), 5′ halves, and 3′ halves [11,12].

Small noncoding RNAs (sncRNAs) are known to play key regulatory roles during embryogenesis across organisms (reviewed by [13]), including regulating maternal-zygotic transitions (e.g., by marking maternal RNA for degradation) [14], maintaining pluripotency [15], and controlling cellular differentiation and fate decision [16]. In cattle, there is a high rate of embryonic mortality during early development, particularly during the first two weeks of gestation [17,18], a period encompassing first lineage segregation for the formation of the inner cell mass (ICM) and the trophectoderm (TE) cells [19], and the onset of conceptus elongation, marked by exponential proliferation of trophoblast cells and substantial production of interferon-tau for maternal recognition of pregnancy [20]. Cells from the ICM are pluripotent and form all three embryonic germ layers (e.g., endoderm, mesoderm, and ectoderm), which form all tissues of the embryo proper. Differentiation of TE cells contributes to the formation of the extraembryonic structures [21]. Notably, sncRNAs appear to play important roles during this developmental window. In bovine embryos specifically, miRNAs appear to be essential for blastocyst formation, as their depletion inhibited morula-to-blastocyst transition [22]. Furthermore, miRNAs play critical roles regulating placental development [23], including regulating key trophoblast functions such as proliferation, migration, invasion, and differentiation in human first-trimester trophoblast cells and murine trophoblast stem cells [24], and contributing to the exponential proliferation of trophoblast cells during conceptus elongation in sheep [25]. Similarly, tRNA-derived fragments have also been shown to be essential for early vertebrate embryogenesis, as knockdown of specific 5′ tRNA-derived fragments in zebrafish resulted in embryonic lethality, with evidence suggesting that this regulatory mechanism is conserved in mammalian cells [26]. Given these critical roles during preimplantation development across species, and the lack of information on the role of sncRNAs during bovine conceptus elongation, the objective of this study was to profile two classes of small noncoding RNAs (miRNAs and tRFs) during the onset of conceptus elongation. The goal was to identify small noncoding RNAs playing important roles during this critical stage of development, which is essential for maternal recognition and pregnancy establishment.

## 2. Materials and Methods

### 2.1. Superovulation for In Vivo Embryo Production

**Figure 3:**
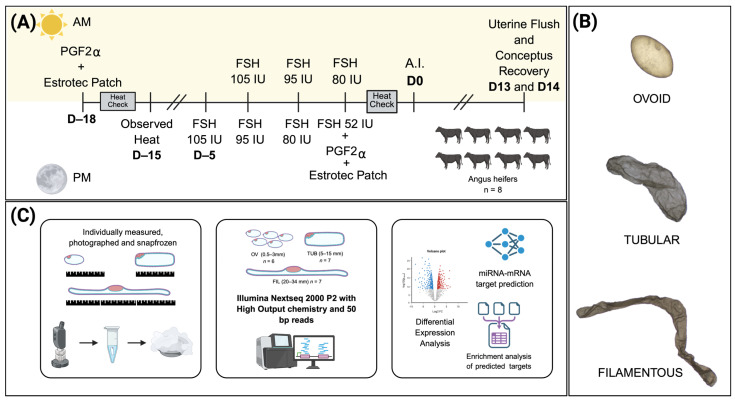
Schematic representation of the superovulation protocol used and experimental procedures employed for transcriptome analyses. (**A**) Eight cyclic Angus heifers were synchronized and superstimulated for embryo collection. On protocol day −18, heifers received an intramuscular injection of PGF_2_α (500 µg cloprostenol sodium; 2 mL Estrumate, Merck Animal Health, Madison, NJ, USA) to induce luteolysis and synchronize estrus, and an Estrotect™ patch was applied for estrus detection. Standing estrus occurred approximately 72 h later (day −15). The superovulation protocol began on day −5 (corresponding to day 10 of the estrous cycle) with the first FSH injection (105 IU; Folltropin, Vetoquinol, Quebec, Canada), followed by decreasing doses administered twice daily on days −4 (95–105 IU), −3 (80–95 IU), and −2 (52–80 IU). On the afternoon of day −2, a second PGF_2_α injection (500 µg cloprostenol sodium) was given concurrently with the final FSH dose, and a new Estrotect™ patch was applied. All heifers exhibited standing estrus within 48 h. Artificial insemination was performed at the onset of estrus (one straw of semen), 12 h later (two straws), and 24 h post-onset (one straw), using semen from a single Bos indicus sire per donor per round. Two proven sires were used across replicates, with each sire assigned to different heifers. (**B**) Representative images of ovoid, tubular and filamentous conceptuses subjected to sequencing. (**C**) Conceptuses recovered from each heifer were individually measured, photographed, and snap-frozen in liquid nitrogen. Ovoid (*n* = 6), Tubular (*n* = 7), and Filamentous (*n* = 7) conceptuses were submitted for small RNA library preparation and sequencing, and subsequent data analysis. Created in BioRender https://BioRender.com/ttswwqe (accessed on 30 March 2026).

Angus heifers were sourced from the Oklahoma State University (OSU) Range Cow Research Center (Stillwater, OK, USA). A trained veterinarian assessed their reproductive tracts using palpation and ultrasonography to confirm cyclicity and ensure normal reproductive tract morphology. Eight pubertal heifers, each with ovaries bearing a corpus luteum, were transported to OSU’s Animal Nutrition and Physiology Center (Stillwater, OK, USA), where they underwent a superovulation protocol. Their estrous cycle was synchronized with an injection of PGF2α (500 µg cloprostenol sodium; 2 mL of Estrumate, Merck Animal Health, Madison, NJ, USA) to induce luteolysis and standing estrus. At the time of PGF2α administration (protocol day −18; Figure 3), an Estrotect™ patch (Estrotect, Rockway, Inc., Spring Valley, WI, USA) was placed midway between the hip and tailhead to facilitate visual detection of estrus. Heifers exhibited standing estrus 72 h following the PGF2α administration (day −15). On the afternoon of day −5 (corresponding to day 10 of the synchronized estrous cycle), donor heifers received their first FSH injection (105 IU; Folltropin, Vetoquinol, Quebec, QC, Canada) to stimulate the development of multiple follicles. Decreasing doses of FSH were given twice daily on days −4 (dose range: 95–105 IU), −3 (dose range: 80–95 IU), and −2 (dose range: 52–80 IU). On the afternoon of the final FSH injection (day −2), heifers also received a PGF2α injection (500 µg cloprostenol sodium; 2 mL of Estrumate, Merck Animal Health, Madison, NJ, USA), and an Estrotect™ patch (Estrotect, Rockway, Inc., Spring Valley, WI, USA) was applied to facilitate estrus detection. All donors exhibited estrus within 48 h following the last PGF2α injection. The insemination protocol consisted of using one straw of semen at the onset of standing estrus, two straws 12 h later, and one straw 24 h post-onset. Four straws of semen from a single *Bos indicus* sire were used per round, ensuring that each heifer received semen exclusively from one bull within that round. Across the experiment, two proven highly fertile sires were utilized, with each sire’s semen assigned to different heifers.

### 2.2. Nonsurgical Uterine Flush

Uterine flushes were performed in the donor heifers on days 13 or 14 post-insemination using a nonsurgical technique optimized for retrieving intact elongating conceptuses. Seven donor heifers were flushed on day 13 and one heifer on day 14 post-breeding (Supplemental Table S1). A two-way Luer-lock catheter (catalog no. 19982/0104; Minitube of America, Verona, WI, USA) was inserted into one uterine horn, and its balloon inflated with flush medium (BioLife ADVANTAGE™ complete flush medium; Agtech, Inc., Manhattan, KS, USA). The horn was then flushed using a 50 mL sterile Air-Tite 2-Part Luer Slip Syringe (Fisher Scientific Ltd., Ottawa, ON, Canada) with a polypropylene barrel and polyethylene plunger. Approximately 50 mL of flush medium was initially introduced into the uterine horn, followed by gentle massage for about 30 s. The flush medium was subsequently retrieved and collected in a glass beaker. This process was repeated, using up to 2 L of flush medium for thoroughly rinsing the horn, and then performed on the contralateral horn. The collected fluid was visually inspected for elongating conceptuses. Next, flushed contents were filtered through a 40 μm nylon mesh filter (Miniflush^®^; Minitube of America, Verona, WI, USA), rinsed with additional flush medium and re-examined for conceptuses.

Recovered conceptuses (*n* = 66) were individually photographed using Leica LAS X software (version 3.7.6) and transferred into 1.5 mL Eppendorf tubes containing 100 μL of flush medium. Following imaging, each conceptus was snap-frozen in liquid nitrogen and stored at −80 °C for RNA isolation.

### 2.3. RNA Isolation and Small-RNA Sequencing

Twenty samples representing the ovoid (OV, *n* = 6; length: 0.5–3 mm), tubular (TUB, *n* = 7; length: 5–15 mm), and filamentous (FIL, *n* = 7; length: 20–34 mm) developmental stages were selected for sequencing. These conceptuses underwent RNA isolation, library preparation, and small RNA sequencing. Due to low RNA yield from a single ovoid conceptus, 2–5 conceptuses were pooled to create the six biological replicates of ovoid conceptuses for sequencing. In contrast, tubular and filamentous conceptuses yielded sufficient RNA for sequencing from individual samples.

Total RNA was extracted using a modified TRIzol–chloroform protocol for phase separation, followed by purification of the aqueous phase with the Zymo Direct-zol RNA MiniPrep Kit (Zymo Research, Irvine, CA, USA) according to the manufacturer’s instructions, as previously described [27]. To eliminate DNA contamination, RNA was treated with DNase I (RNase-Free DNase Set; Qiagen, Hilden, Germany) during extraction. Finally, total RNA was eluted in 200 μL of DNase/RNase-free water and stored at −80 °C until sequencing.

Before sequencing, quality control procedures were conducted. RNA concentrations were determined using fluorometric analysis with a Thermo Fisher Qubit fluorometer. The overall RNA quality was assessed using an Agilent Tapestation instrument. Following initial QC steps, sequencing libraries were generated using the QIAseq miRNA library prep kit according to the manufacturer’s protocol. Briefly, a pre-adenylated DNA adapter was ligated to the 3′ end of the small RNA molecules, followed by RNA adapter ligation to the 5′ end. cDNA synthesis was then performed using a first-strand primer containing a unique molecular index. After a magnetic bead-based cleanup of the cDNA, the library was amplified with primers giving each library a unique multiplexing index. Final libraries for each sample were assayed on the Agilent Tapestation for the appropriate size and quantity. These libraries were then pooled in equimolar amounts as ascertained via fluorometric analyses. Final pools were absolutely quantified using qPCR on a Roche LightCycler 480 instrument with Kapa Biosystems Illumina Library Quantification reagents. Sequencing was performed using custom primers on an Illumina NextSeq 2000 (P2 flow cell; Illumina, San Diego, CA, USA) instrument with High Output chemistry and 50 bp pair-ended reads.

### 2.4. Processing and Alignment of Small RNAseq Data

FastQC [28] was used to evaluate the quality of the raw sequences, and cutadapt (version 2.10) was used to remove the adapter sequence (-a AACTGTAGGCACCATCAAT) [29]. Quality trimming was performed using SolexaQA++ (version 3.1.7.1), DynamicTrim (Phred score ≥ 19), and LengthSort functions (Length ≥ 13 for tRFs and ≥ 17 for miRNAs) to obtain clean reads [30]. Abundance estimation of known miRNAs was performed using the bovine reference genome (ARS-UCD1.2) [31], miRDeep2 (version 0.1.2) modules, and miRbase (Release 22.1) [32]. Briefly, the clean reads were aligned to the reference genome using the mapper.pl with adjusted parameters (-d -c -j -l 17 -m -p -u -v -i), and the collapsed reads aligned with bowtie (version 1.3.1) [33]. The file used to generate miRNA counts was free of mismatches and aligned to known bovine precursor and mature miRNA sequences from miRbase using the quantifier.pl module with adjusted parameters (-p -m -r -t bta -g 0).

The tRNA fragment prediction was carried out using the MINTmap pipeline [34]. Genomic tRNA sequences were obtained from gtrnadb (http://gtrnadb.ucsc.edu, accessed on 12 December 2023) and mitotRNAdb (http://mttrna.bioinf.uni-leipzig.de, accessed on 12 December 2023). Custom scripts were used to remove introns, add discriminator bases at the −1 position, and add CCA tails to each tRNA sequence. Potential tRF sequences were generated by using a sliding window to break each mature tRNA sequence into lengths ranging from 15 to 60 nucleotides. MINTmap then aligned processed reads to a look-up table of the candidate tRF sequences exclusively located in regions associated with annotated tRNAs. The raw counts of exclusively mapped tRFs were kept for further analysis.

### 2.5. Differential Expression in miRNA and tRF According to Stage of Development

Elongating conceptuses sequenced in this study originated from multiple donors (*n* = 8) and sires (*n* = 2), with ovoid conceptuses pooled to yield sufficient total RNA for small RNA sequencing. Sample pooling was accounted for in the metadata, with the Donor and Sire columns reflecting unique identifiers and combinations (e.g., ‘23_48’ for Donor; ‘Gus_and_SuperC’ for Sire) that indicate pooled samples within the dataset.

To disentangle the effects of developmental stage (Ovoid, Tubular, Filamentous) from potential confounding variables (donor and sire), differential expression modeling and normalization were first performed using the DESeq2 (v 1.48.1) package [35] in R (v 4.5.1). A DESeqDataSet was constructed from the raw count matrices with sample metadata, using the design formula ~ Donor_Sire + Stage, where Donor_Sire is a composite factor combining Donor and Sire (resulting in 8 unique levels). This design accounted for combined donor-sire variation while evaluating stage-related differences. The DESeq function was applied to estimate size factors and dispersions. To stabilize variance and handle zero counts, Variance Stabilizing Transformation (VST) was then applied with blind = FALSE to incorporate the experimental design, yielding stabilized counts. These VST-normalized counts were further corrected for Donor_Sire batch effects using removeBatchEffect from the limma package (v 3.64.3) [36], while preserving Stage effects via the design matrix ~ Stage. The corrected VST counts were saved for downstream use.

Principal Component Analysis (PCA) was performed on the transposed corrected VST-normalized counts to identify primary sources of variability in miRNA and tRF expression. Columns with zero variance were removed to ensure robustness, and PCA was conducted using prcomp with scaling enabled to calculate principal component (PC) scores. To visualize the distribution of samples from the Ovoid, Tubular, and Filamentous stages in the context of the primary sources of miRNA and tRF expression variability, PCA scatterplots were generated with PC1 and PC2 as axes for the miRNA and tRF datasets, where points were colored according to developmental stage.

To quantify the influence of metadata variables (Stage, Donor, and Sire) on miRNA and tRF expression variability, we employed the Random Forest (v 4.7–1.2) algorithm [37] implemented in R (v 4.5.1) as a machine learning approach. Regression models were constructed for the first 20 PCs (explaining 100% of the total variance), with the predictor dataset including Stage, Donor, and Sire, and separate models fitted for each PC. Feature importance, quantified as IncNodePurity, was extracted from each model. IncNodePurity measures the reduction in node impurity attributable to each metadata variable across decision trees, with higher values indicating greater explanatory power for the target principal component. Importance values were aggregated across the 20 PCs using variance-weighted averaging, where IncNodePurity was scaled by the proportion of variance explained by each PC. The variance-weighted importance was reported to reflect proportional contributions to the overall dataset variability.

Random Forest regression analyses revealed a significant influence of donor and sire on miRNA and tRF expression variability. To focus on identifying small RNAs associated with developmental stages (Ovoid, Tubular, Filamentous), differential expression analysis was performed on both datasets while controlling for these donor-sire effects. Pairwise comparisons between developmental stages (Ovoid vs. Tubular, Ovoid vs. Filamentous, and Tubular vs. Filamentous) were conducted using the results function in DESeq2 with specified contrasts, employing the Wald test to assess differential expression. miRNA and tRF with an adjusted *p*-value < 0.05 were classified as significantly differentially expressed.

### 2.6. In Silico Prediction of miRNA-mRNA and tRF-mRNA Targets

The miRanda algorithm (version 3.3a) [38] was employed to predict miRNA-mRNA target relationships for the differently abundant miRNAs. miRanda predicts interactions between miRNAs and the 3′ untranslated regions (3′ UTRs) of target genes by evaluating seed sequence complementarity and the thermodynamic stability of the resulting miRNA-mRNA duplex [38]. For this analysis, the following adjusted parameters were used (-sc 150 -en -20 -strict) to enhance sensitivity in target prediction. All bovine protein-coding genes’ 3′ UTR sequences were obtained using the Ensembl BioMart tool (Release 100) [39]. The resulting predicted targets were then used for downstream functional analysis.

### 2.7. Gene Ontology (GO) Analysis of the miRNA

To better understand specific biological functions of the differently abundant miRNA targeted genes in the predictive framework, gene ontology (GO) analysis was performed using ShinyGO 0.80 (https://bioinformatics.sdstate.edu/go80/, accessed on 17 March 2025) [40]. For this analysis, the list of candidate gene targets identified by miRanda of upregulated miRNAs was used, and all expressed protein-coding genes were used as the background gene set. KEGG, Molecular Function, Cell Component, and Biological Process pathway enrichment analysis were performed using the ShinyGO 0.80 [40]. The enrichment of GO terms was identified with FDR and a cut-off *p*-value of 0.05.

## 3. Results

### 3.1. Conceptus Recovery

**Figure 4:**
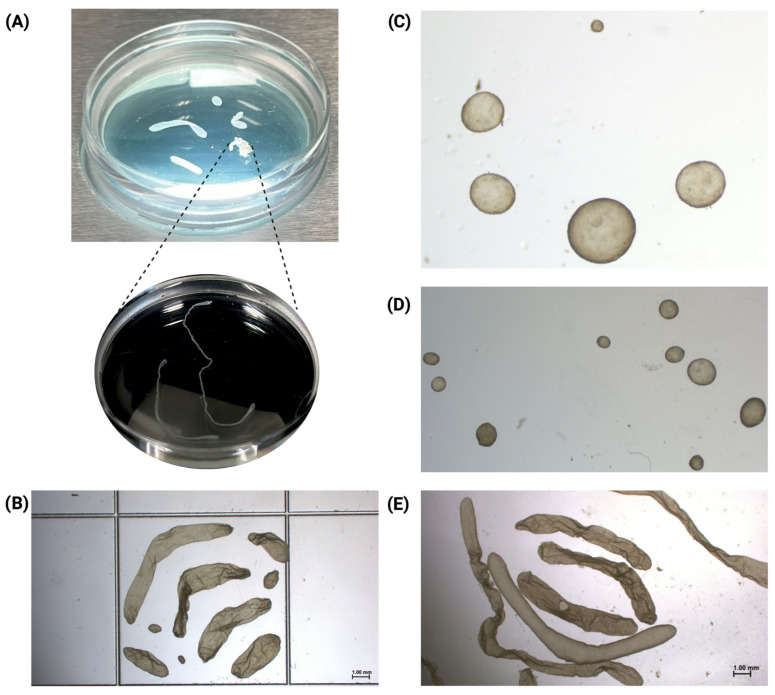
Heterogeneous conceptus recovery within donors flushed at days 13 and 14 post-insemination. (**A**) Represents a uterine flush from donor 386 (D13) in a 35 mm Petri dish without the use of a stereoscope. It illustrates the recovery of six conceptuses displaying mixed developmental stages. The inset (dashed lines) highlights two elongated filamentous conceptuses after gentle detangling. (**B**) Conceptus recovered from donor 9008 (D14), showing ovoid and tubular structures recovered. Of note, only tubular conceptuses from donor 9008 were submitted for sequencing analysis. (**C**) Spheroid and ovoid stage conceptuses recovered from donor 23 (D13). (**D**) Spheroid and ovoid stage conceptuses recovered from donor 48 (D13). (**E**) Tubular and Filamentous conceptuses recovered from donor 375 (D13). Scale bars = 1.00 mm.

A total of 66 conceptuses were recovered across eight donors, ranging from 2 to 14 conceptuses per donor (mean ± SD: 8.24 ± 3.88). Detailed information on conceptus recovery and stage distribution per donor and sire is provided in Supplemental Table S1. Seven donor heifers were flushed on days 13 and one heifer on day 14 post-insemination following the superovulation protocol. Notably, ovoid conceptuses were recovered from both day 13 and day 14 flushes. Within individual donors, conceptus development was not always homogeneous; some donors yielded mixed populations comprising ovoid, tubular, and filamentous conceptuses (donor 386/ Figure 4A), while others yielded ovoid and tubular conceptuses (donors 48, 406, and 9008; Figure 4B), exclusively ovoid or earlier spheroid conceptuses (donors 23, 397, and 8028; Figure 4C,D), or tubular and filamentous conceptuses exclusively (donor 375; Figure 4E), reflecting natural variation in developmental progression following superovulation.

As for the sire composition of the conceptuses that were sequenced, the two sires were represented across samples from tubular and filamentous conceptuses. Additionally, because the biological replicate for the ovoid stage contained pools of ovoid conceptuses, there were both samples of ovoid conceptuses originating from a single sire, or samples with pooled ovoid conceptuses from two sires (Supplemental Table S1).

### 3.2. Principal Component and Random Forest Analyses

**Figure 5:**
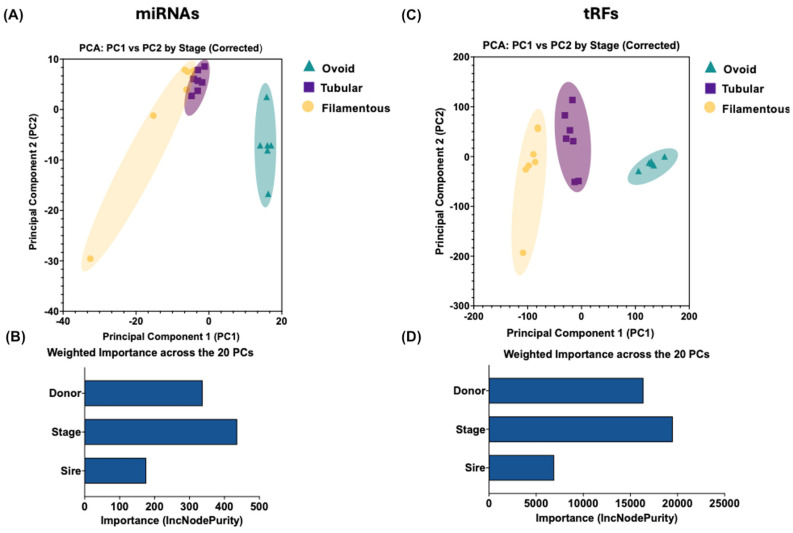
Principal component analysis and random forest feature importance for stage-associated variability in miRNA and tRF expression. (**A**,**C**): PCA scatterplots of VST-normalized counts for miRNA (**A**) and tRF (**C**), corrected for donor and sire effects. Samples are plotted on PC1 and PC2, colored by developmental stage (Ovoid, Tubular, Filamentous). In the PCAs, colors and shapes denote developmental stage: ovoid conceptuses (green triangles), tubular conceptuses (purple squares), and filamentous conceptuses (yellow circles). (**B**,**D**): Variance-weighted IncNodePurity from Random Forest regression on the first 20 PCs for miRNA (**B**) and tRF (**D**). Bars show the relative contributions of Stage, Donor, and Sire to expression variability, with Stage as the primary factor (miRNA: 436.66; tRF: 19,503.60), followed by Donor (miRNA: 337.80; tRF: 16,388.92) and Sire (miRNA: 176.32; tRF: 6922.02).

Principal component analysis (PCA) of variance-stabilizing transformation (VST)-normalized counts, corrected for combined donor and sire effects, demonstrated clear separation of samples by developmental stage. Specifically, ovoid conceptuses clustered distinctly from tubular and filamentous samples in both the miRNA (Figure 5A) and tRF (Figure 5C) datasets, highlighting stage-associated expression patterns.

To quantify the relative contributions of metadata variables (stage, donor, and sire) to the variability in small RNA expression, Random Forest regression models were applied to the first 20 principal components (PCs), which captured 100% of the total variance in each dataset. For miRNAs, variance-weighted IncNodePurity values identified stage as the predominant factor (436.66), followed by donor (337.80) and sire (176.32; Figure 5B). Similarly, for tRFs, stage was the leading contributor (19,503.60), closely followed by donor (16,388.92) and sire (6922.02; Figure 5D).

Based on these findings, differential expression analyses for both miRNA and tRF datasets were designed to account for the combined donor and sire effects, ensuring accurate identification of stage-associated expression differences.

### 3.3. Differential Expression Analyses

***miRNAs***. A total of 455 unique miRNAs were detected across 20 bovine embryo samples (Ovoid, *n* = 6; Tubular, *n* = 7; Filamentous, *n* = 7), totaling 31.14 million reads (Supplemental Table S2). Stage-specific sums were 3.1 million (Ovoid), 17.2 million (Tubular), and 10.8 million (Filamentous). Per-sample read counts ranged from 152,189 to 3,430,632 (median: 1,422,406). Median expression per miRNA was 211 reads, with the top 10% of miRNAs (*n* = 45) comprising 96.1% of reads.

After correcting for donor and sire effects, the transition between the Ovoid and Tubular stages revealed 6 differently expressed miRNAs (padj < 0.05; Table 1). All exhibited higher expression in Ovoid conceptuses, with log2 fold changes ranging from 2.7 to 7.3, corresponding to approximately 6- to 160-fold increases. Strikingly, among the six differentially expressed microRNAs, four were members of the let-7 family (bta-let-7g, bta-let-7f, bta-let-7a-5p, and bta-let-7c), suggesting an important role of this family of miRNAs in regulating this initial transition. Outside the let-7 family, bta-miR-224 (log2FC = 2.67) was also significantly upregulated in Ovoid versus Tubular (padj < 0.05). Furthermore, there were 3 differently abundant miRNAs between the ovoid and filamentous transition, including 2 miRNAs from the let7 family (bta-let-7g, and bta-let-7f; Log2FC = 3.81–4.12) and bta-miR-449a (Log2FC = 7.82). Indeed, bta-miR-449a was also significantly upregulated in the Ovoid versus Tubular comparison (log2FC = 7.32), indicating its consistent enrichment in Ovoid conceptuses across both transitions.

Of note, the differentially abundant miRNAs were among the most highly expressed in our dataset. For instance, bta-let-7a-1 and bta-let-7f-2 ranked within the top 10% of all detected miRNAs based on read counts, bta-let-7g and bta-let-7c within the top 20%, and bta-miR-224 and bta-miR-449a within the top 30%. Their high expression further underscores their potential biological relevance during conceptus elongation.

***tRFs*.** Regarding the tRF data generated (Supplementary Table S3), a total of 40,588 unique tRFs were detected across the 20 samples (Ovoid, *n* = 6; Tubular, *n* = 7; Filamentous, *n* = 7), totaling 4.48 million reads. Stage-specific sums were 0.90 million (Ovoid), 2.24 million (Tubular), and 1.32 million (Filamentous). Per-sample read counts ranged from 29,833 to 583,359 (median: 176,066). Median expression per tRF was 3 reads, with the top 10% of tRFs (*n* = 4058) comprising 95.3% of reads.

Contrary to our initial hypothesis that tRFs would be differentially expressed across developmental stages, the differential abundance analysis, after adjusting the DESeq2 models for donor and sire effects, revealed no differently abundant tRFs among ovoid, tubular, and filamentous conceptuses.

**Figure 6:**
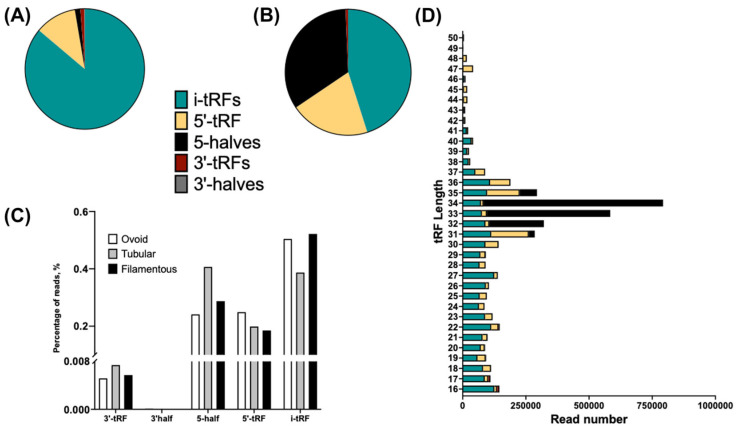
tRF subtype composition, abundance, and length distribution in bovine conceptuses during elongation. (**A**) Distribution of tRF subtypes among the 40,588 unique tRFs detected, showing that 86.1% were i-tRFs, 11.2% were 5′-tRFs, 1.5% were 5-halves, 1.1% were 3′-tRFs, and 0.1% were 3′-halves. (**B**) Proportion of total reads assigned to each tRF subtype. Less than 1% of reads corresponded to 3′-tRFs and 3′-halves, whereas 45.1% were i-tRFs, 33.8% were 5-halves, and 20.5% were 5′-tRFs. (**C**) Proportion of reads assigned to each tRF subtype observed in Ovoid, Tubular and Filamentous conceptuses. (**D**) Length distribution of tRFs, ranging from 16 to 50 nucleotides. More than half of all reads (55%) were concentrated between 31 and 36 nt, with a prominent peak at 34 nt (17.7%, 793,049 reads), followed by 33 nt (13.1%), 32 nt (7.2%), and 35 nt (6.6%). Shorter fragments (16–30 nt) each accounted for less than 3.3% of reads, while longer fragments (>36 nt) were rare (<1%), indicating strong enrichment for tRFs in the 31–36 nt range.

Given that distinct tRF subtypes can exert different biological functions [41] and exhibit variable lengths across tissues [8], Figure 6A–D presents novel data of tRF expression patterns in bovine conceptuses during elongation. Notably, among the 40,588 unique tRFs detected, 86.1% were i-tRFs, 11.2% were 5′-tRFs, 1.5% were 5-halves, 1.1% were 3′-tRFs, and 0.1% were 3′-halves (Figure 6A). In terms of read abundance, less than 1% of the total reads corresponded to 3′-tRFs and 3′-halves, whereas 45.1% were i-tRFs, 33.8% were 5-halves, and 20.5% were 5′-tRFs (Figure 6B). Differential expression analysis using DESeq2 revealed no significant differences in the abundance of tRF subtypes across developmental stages (Figure 6C). Regarding tRF length distribution (Figure 6C), it ranged from 16 to 50 nucleotides, with 55% of the 4,475,231 total reads concentrated between 31 and 36 nt, peaking at 34 nt (17.7%, 793,049 reads), followed by 33 nt (13.1%, 584,047 reads), 32 nt (7.2%, 321,232 reads), and 35 nt (6.6%, 294,131 reads). Shorter fragments (16–30 nt) each contribute less than 3.3%, with minor peaks at 22, 27, and 30 nt, while longer fragments (>36 nt) drop significantly, mostly below 1%, indicating a strong enrichment for tRFs in the 31–36 nt range.

### 3.4. Functional Analyses of the Significant miRNAs

**Figure 7:**
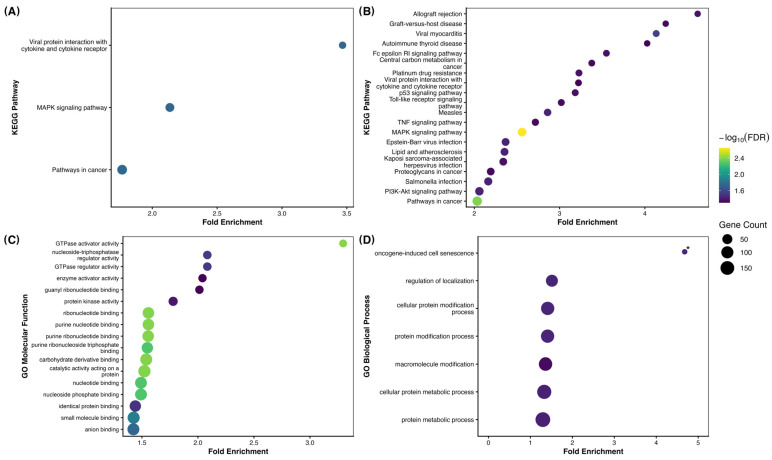
Functional enrichment analysis of target genes predicted for differentially expressed miRNAs. (**A**) KEGG pathways enriched among 830 genes targeted by DE miRNAs distinguishing ovoid (OV) and tubular (TUB) conceptuses. (**B**) KEGG pathways enriched among 621 genes targeted by DE miRNAs between ovoid (OV) and filamentous (FIL) conceptuses. (**C**) Gene Ontology (GO) Molecular Function terms enriched for target genes of DE miRNAs identified in the OV vs. FIL comparison. (**D**) Gene Ontology (GO) Biological Process terms enriched for target genes of DE miRNAs identified in the OV vs. FIL comparison. For all panels, the x-axis represents fold enrichment (observed/expected gene ratio). Dot size indicates the number of genes associated with each term, and color represents statistical significance expressed as −log_10_ of the false discovery rate (FDR), adjusted using the Benjamini–Hochberg method. In panel (**D**), the asterisk (*) denotes a term with fold enrichment greater than 20, which exceeds the displayed x-axis range and is therefore truncated for visualization.

In total, 1260 potential target genes were predicted using miRanda for the DE miRNAs (Supplementary Table S4). Specifically, the mature forms of the six DE miRNAs distinguishing OV from TUB conceptuses were predicted to regulate 830 unique genes (Supplementary Table S5). Functional enrichment of this gene set revealed three significantly enriched KEGG pathways (FDR < 0.05): MAPK signaling (30 genes), Viral protein interaction with cytokines and cytokine receptors (12 genes), and Pathways in cancer (44 genes) (Supplementary Table S6; Figure 7A).

Enrichment analysis of 621 unique genes targeted by bta-let-7g, bta-let-7f, and bta-miR-449a, the differently abundant miRNAs between the OV vs. FIL comparison (Supplementary Table S7), identified 20 significantly enriched KEGG pathways (FDR < 0.05, Supplementary Table S8; Figure 7B). Key pathways include MAPK signaling (27 genes, FDR = 0.002) and PI3K-Akt signaling (24 genes, FDR = 0.037), which are critical for cell proliferation, survival, and apoptosis. Pathways in cancer (38 genes, FDR = 0.004) and p53 signaling (9 genes, FDR = 0.044) underscore roles in oncogenesis and tumor suppression. Immune-related pathways, such as Toll-like receptor signaling (9 genes, FDR = 0.046), TNF signaling (11 genes, FDR = 0.046), and Fc epsilon RI signaling (8 genes, FDR = 0.044), Autoimmune thyroid disease (6 genes, FDR = 0.049), highlight roles in immune response and inflammation regulation. There were also enriched pathways associated with metabolism, such as Lipid and atherosclerosis (17 genes, FDR = 0.037) and Central carbon metabolism in cancer (8 genes, FDR = 0.046).

For Molecular Function, enrichment analysis revealed 17 significant pathways, with overrepresentation of terms related to nucleotide binding, including purine nucleotide binding, ribonucleotide binding, and ribonucleoside triphosphate binding. These categories encompass proteins that selectively interact with molecules such as ATP and GTP, which are critical for energy transfer, enzymatic activity, and intracellular signaling. Additional enriched terms included protein kinase activity and catalytic activity, reflecting the importance of phosphorylation and enzymatic regulation, as well as functions linked to the regulation of small GTPases (activator and regulator activity), highlighting roles in signal transduction and cytoskeletal dynamics. Broader categories such as protein binding, anion binding, and enzyme activator activity were also significantly enriched (Supplementary Table S9; Figure 7C). For Biological Process, enrichment analysis revealed 7 significant pathways, highlighting several mechanisms tied to protein regulation and modification, cellular organization, and oncogene-induced cell senescence (Supplementary Table S10; Figure 7D).

## 4. Discussion

**Figure 8:**
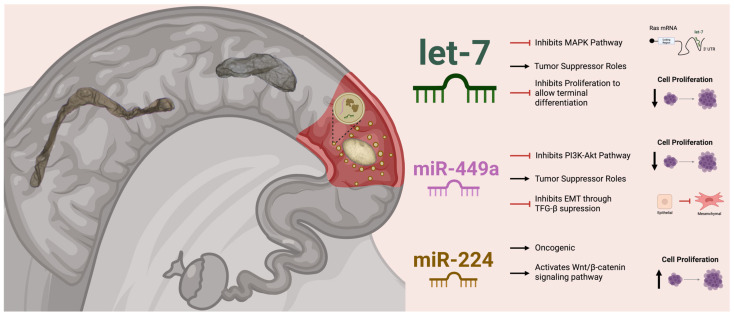
Proposed mechanism of action of the differently abundant miRNA. Created in BioRender (https://BioRender.com/6s9q7t4, accessed on 30 March 2026).

The present study aimed to profile two classes of small non-coding RNAs (miRNA and tRFs) during bovine conceptus elongation to gain insights into their function during this critical period for establishing pregnancy. For that, ovoid, tubular, and filamentous conceptuses were subjected to small RNA sequencing. Interestingly, after correcting for donor and sire effects, the let-7 family of miRNAs emerged as potential regulators of the initial transition between ovoid and tubular stages. Given the prominence of let-7 in our differential expression and enrichment analyses and its well-characterized function in other organisms, we further explored its role to provide insights into its potential regulatory contributions during bovine conceptus elongation. A hypothetical model for the putative role of let-7 and two additional miRNAs (miR-449a and miR-224), which are candidate regulators of proliferation during bovine conceptus elongation, is summarized in Figure 8.

As an overview of the family of let-7 miRNAs, starting with the name, let-7 is derived from “lethal-7”, terminology gained from its discovery in the nematode *Caenorhabditis elegans*, where mutations in this gene were found to cause lethal developmental defects. In fact, let-7 and another miRNA, lin-4, were the first two miRNAs ever discovered, and let-7 was the first miRNA reported in humans [42]. Multiple paralogs of the let-7 family of miRNAs are often observed across organisms [42]. The bovine genome (Bos taurus, ARS-UCD1.2) contains 11 copies of let-7 family genes (let-7a-1, a-2, a-3, b, c, d, e, f-1, f-2, g, i). These are precursor miRNA genes, scattered across chromosomes 5, 8, 11, 15, 18, 22, and X [32]. Unfortunately, the present study does not allow us to dissect potential functional redundancy or specialized roles for different let-7 variants. The fact that the let-7 genes are scattered across multiple chromosomes suggests that their expression may be independently regulated by distinct genomic regulatory elements (e.g., promoters, enhancers). This distribution could also be associated with cell lineage-specific expression during preimplantation development.

Regarding the known function of let-7 miRNA, in *C. elegans*, it was found to regulate larval differentiation by targeting the lin-41 mRNA [43]. The protein encoded by the lin-41 gene promotes the proliferation of seam cells (a hypodermal stem cell-like cell) and inhibits their differentiation. By repressing lin-41, let-7 allows seam cells to exit the proliferative state and undergo terminal differentiation into adult hypodermal cells. A similar mechanism of action is observed in mice during embryonic development, where let-7 is downregulated in favor of proliferation during the first few divisions and upregulated around the morula stage to allow terminal differentiation for first lineage segregation (e.g., ICM and TE). For instance, two members of the let-7 family (let-7a and let-7g) were found to decrease drastically between the zygote and two-cell stages of the mouse embryo, and their expression remained low up to the 8-cell stage, increasing at the morula stage. Upon the formation of the blastocyst, let-7 expression was higher in the ICM compared to the trophectoderm cells [44]. Collectively, these studies highlight the conserved role of let-7 miRNAs in inhibiting cellular proliferation in favor of terminal differentiation.

In highly proliferative cells, let-7 expression is actively suppressed by the RNA-binding protein LIN28, which represses let-7 biogenesis through direct binding to let-7 precursor transcripts, thereby promoting proliferation [45]. The LIN-28-let-7 miRNA axis play important roles in placental development across species [23]. In immortalized first-trimester human trophoblast cells, knockdown of *LIN28* results in increased let-7 expression accompanied by reduced expression of genes associated with proliferation, and reduced cell proliferation [46]. Importantly, functional studies in sheep (a species that, like cattle, undergoes conceptus elongation prior to implantation) have demonstrated a direct role for the LIN28-let-7 axis in trophoblast development. Lentiviral-mediated shRNA knockdown of paralogs *LIN28A* and *LIN28B* in day 9 hatched sheep blastocysts, specifically targeting trophectoderm cells, impaired conceptus elongation by day 16 of gestation (e.g., resulted in shorted conceptuses). As expected, expression of let-7 miRNAs was increased in day 16 conceptuses following *LIN28A* and *LIN28B* knockdown [25]. Transcriptomic analyses of these conceptuses revealed enrichment of biological pathways essential for placental and fetal development [47]. Taken together, the regulatory role of let-7 observed in *C. elegans* during larval development, in mice during early embryogenesis, and in sheep during conceptus elongation parallels our findings during bovine conceptus elongation. In the present study, the earliest conceptus stage analyzed (ovoid) exhibited higher abundance of let-7 miRNAs. Elevated let-7 levels in ovoid conceptuses are consistent with a hypothesized role in favoring early differentiation while restraining proliferation, analogous to its documented functions in other organisms. Conversely, during the rapid elongation phases in tubular and filamentous stages, reduced let-7 expression aligns with the need for high cellular proliferation, perhaps more so in trophectoderm cells, enabling the exponential growth required for proper placenta formation and maternal recognition of pregnancy. However, whether let-7 directly drives or merely associates with these proliferative dynamics during bovine preimplantation development remains to be determined through functional studies.

In cancer, let-7 has also been observed to play a role favoring differentiation. In Type I tumor cell lines, which are less differentiated and have a mesenchymal phenotype (indicative of a more advanced cancer), let-7 is almost absent [48]. However, in Type II lines, which are more differentiated and present an epithelial phenotype (indicating a less advanced cancer), the expression of let-7 is greater [49]. Let-7 miRNAs are known as tumor suppressors that inhibit the MAPK/ERK pathway (also known as the Ras-Raf-MEK-ERK), a critical pathway inducing cellular proliferation [50]. The 3′ untranslated regions (3′ UTRs) of human RAS genes include several let-7 complementary sites (LCSs), allowing let-7 to influence RAS expression [51]. In the present study, our functional enrichment KEGG analysis of the targeted genes of let-7 reflected their significant role in regulating MAPK signaling. In addition to MAPK, the PI3K-Akt signaling pathway, another pathway inducing cellular proliferation, was enriched in the analyses of the targeted genes from the differentially abundant miRNA between OV and FIL conceptuses. Notably, miR-449a has been shown to inhibit the PI3K-Akt pathway through activation of the p53 pathway in breast cancer [52] and to exert tumor-suppression roles in cancer of glial cells [53].

Furthermore, miR-449a can suppress the transforming growth factor-beta (TGF-β), a cytokine that plays a major role in cell growth, differentiation, apoptosis, and cellular immunity [54]. Particularly, miR-449a has been found to repress TGF-β-mediated epithelial–mesenchymal transition (EMT), which in cancer is associated with progression, metastasis, and increased tumor invasiveness [55]. During embryogenesis, the epithelial cells (tightly packed, adherent, expressing adhesion molecules, and organized in a sheet-like architecture) undergo EMT, an essential process for gastrulation, which entails the differentiation of ectoderm, mesoderm, and endoderm from the simple epithelial epiblast [56]. During gastrulation, EMT enables epiblast cells to downregulate cell adhesion molecules, such as E-cadherin, thereby acquiring migratory and invasive properties. These changes facilitate the dynamic cell movements necessary for the formation of the three germ layers [57]. In cattle, gastrulation is initiated around days 11–12 of embryogenesis [58]. Because cows in the present study were flushed on days 13–14, the higher expression of miR-449a in ovoid conceptuses (the earliest stage sampled) is consistent with a potential role of miR-449a regulating differentiation around the time of gastrulation in cattle. Additionally, miR-449a has been suggested to play a role in regulating endometrial receptivity in goats [59], raising the possibility that its elevated abundance in ovoid conceptuses may similarly contribute to the modulation of endometrial receptivity at the onset of conceptus elongation in cattle.

The last differently abundant miRNA that increased in ovoid compared to tubular conceptuses, miR-224, has been described as an oncogenic miRNA, facilitating proliferation and invasion of cancer cells [60,61]. Notably, miR-224 can activate the Wnt/β-catenin signaling pathway by binding to the 3′-UTR of targeted genes (*GSK3β* and *SFRP2*), inducing progression and metastasis of colorectal cancer [62]. The Wnt/β-catenin pathway is active during preimplantation development in the mouse, exerting dual roles in embryonic stem cells, by promoting differentiation when activated as part of the Wnt/β-catenin signaling pathway, and inducing stable pluripotency independently of signaling [63]. The observed contrasting functions regulating cellular proliferation between miR-224 and both let-7 and miR-449a could reflect cell type (e.g., tissue)-specific expression, where, for instance, proliferation is needed for expansion of one cell lineage (trophoblast cells), but suppression is needed for terminal differentiation of another (embryonic disc).

The enrichment of immune-related pathways among the target genes of differently abundant miRNAs between the ovoid and filamentous transition could be related to the role of EVs regulating endometrial receptivity for implantation. As miRNAs (including let-7) have been identified as cargo of extracellular vesicles (EV) from the bovine oviduct and uterus [64], and conceptus secreted EVs can be up taken by the uterus [65], it is possible that EVs secreted by ovoid conceptus could enhance local endometrial receptivity favoring implantation (Figure 8).

The present study failed to identify significant differences in tRFs across the distinct developmental stages sequenced. Although once regarded merely as degradation intermediates or by-products of random cleavage of tRNAs, tRFs are now increasingly recognized as a distinct class of small RNAs with potential regulatory functions [66]. Recent research provided evidence of their biological functions on humans and animals and their association with disease states, including cancer [9,10]. In cancer, tRFs have been shown to suppress cell proliferation. For example, in breast cancer, select tRFs can bind to the YBX1 protein, which normally promotes proliferation by stabilizing oncogenic transcripts. By competing with these transcripts for YBX1 binding, these select tRFs inhibit cancer cell proliferation [67]. In cattle, dysregulation of tRF expression has been associated with the Large Offspring Syndrome (LOS), a congenital overgrowth condition often occurring in offspring produced using assisted reproductive technologies [8]. Given their potential role in regulating cellular proliferation, we hypothesized that tRFs could also contribute to processes occurring during conceptus elongation. Although no differentially abundant tRFs were detected among ovoid, tubular, and filamentous stages in the present study, this work provides the first comprehensive profiling of tRFs during this pivotal period of pregnancy.

A limitation of the present study is the modest sample size, with 20 conceptuses distributed across three developmental stages and eight donors, which constrains statistical power. Nevertheless, the miRNAs identified as differentially abundant, particularly the let-7 family members, bta-miR-449a, and bta-miR-224, ranked among the most highly expressed miRNAs in the dataset, suggesting the observed signals reflect genuine biological differences rather than marginal statistical artifacts. The modest statistical power may have limited our ability to detect differential abundance among lowly expressed miRNAs, and future studies with larger sample sizes would provide a more complete picture of sncRNAs dynamics during bovine conceptus elongation. Additionally, the present study subjected the donor heifers to a superovulation protocol, which has consequences on oocyte quality [68] leading to reduced fertilization rates [69], altered oviduct [70] and endometrial function [71], and reduced competence for preimplantation development as compared to naturally ovulating females [72]. Furthermore, ovarian superstimulation is associated with alterations in blastocyst gene expression compared with that of naturally ovulating females, which could extend into the elongation phase [72]. The presence of multiple conceptuses simultaneously developing within the uterine environment also raises the possibility of embryo-embryo communication influencing individual conceptus development [73]. Future studies comparing sncRNA profiles from superovulated versus naturally cycling donors would help clarify the extent to which these factors contribute to the expression variability observed.

## 5. Conclusions

This study provides the first integrated characterization of miRNAs and tRFs during bovine conceptus elongation. We identified members of the let-7 family as candidate miRNAs associated with the balance between proliferation and differentiation at the onset of conceptus elongation. In particular, elevated let-7 abundance in ovoid conceptuses is consistent with a hypothesized role in promoting terminal differentiation preceding the extensive proliferation required for elongation, analogous to its documented functions in other species. Furthermore, based on their differential abundance and known functions in other biological contexts, miR-449a and miR-224 are hypothesized to play tumor-suppressive and proliferative roles, respectively, at the onset of elongation, potentially reflecting roles in distinct cell lineages undergoing either proliferation or terminal differentiation. Although no stage-dependent differences in tRF abundance were observed, their known functions in other biological systems warrant future investigation of their potential regulatory roles in bovine embryogenesis. Collectively, our findings expand current knowledge of small noncoding RNA dynamics during a critical window of bovine pregnancy and provide a foundation for future mechanistic studies to clarify their contributions to conceptus development and implantation.

## Figures and Tables

**Table 1:** Differentially expressed miRNAs during conceptus elongation.

miRNA ID	Sequence	log2FC	Adj *p*-Value
*Ovoid* vs. *Tubular*			
bta-let-7g	TGAGGTAGTAGTTTGTACAGTT	3.78	0.004
bta-let-7f	TGAGGTAGTAGATTGTATAGTT	3.86	0.011
bta-let-7a-5p	TGAGGTAGTAGGTTGTATAGTT	3.57	0.022
bta-let-7c	TGAGGTAGTAGGTTGTATGGTT	3.37	0.042
bta-miR-449a	TGGCAGTGTATTGTTAGCTGGT	7.32	0.042
bta-miR-224	CAAGTCACTAGTGGTTCCGTTTA	2.67	0.043
*Ovoid* vs. *Filamentous*			
bta-let-7g	TGAGGTAGTAGTTTGTACAGTT	4.12	0.004
bta-let-7f	TGAGGTAGTAGATTGTATAGTT	3.81	0.039
bta-miR-449a	TGGCAGTGTATTGTTAGCTGGT	7.82	0.039

**Note:** Italicized comparison labels (Ovoid vs. Tubular and Ovoid vs. Filamentous) are used solely for visual separation of differentially expressed miRNAs corresponding to each pairwise comparison.

## Data Availability

The sequencing data underlying this article are available in the NCBI Sequence Read Archive (SRA) under BioProject PRJNA1335270.

## Supplementary Materials

The following supporting information can be downloaded at: https://www.mdpi.com/article/10.3390/ani16081181/s1, Table S1: Summary superovulation results; Table S2: miRNA; Table S3: tRF; Table S4: miranda results; Table S5: Target genes of the DE miRNA between OV and TUB; Table S6: EnrichedPathways_KEGG_OVvsTUB; Table S7: Unique targets OV vs. FIL; Table S8: EnrichedPatways KEGG_OVvsFIL; Table S9: Molecular Function_OVvsFIL; Table S10: Biological Process _OVvsFIL.

## Abbreviations

The following abbreviations are used in this manuscript:

3′UTR	3′Untranslated Region
AGO	Argonaute
AI	Artificial Synchronization
cDNA	Complimentary DNA
DEG	Differentially Expressed Gene
DNA	Deoxyribonucleic Acid
EMT	Epithelial-to-Mesenchymal Transition
EV	Extracellular Vesicle
FDR	False Discovery Rate
FIL	Filamentous
FSH	Follicle Stimulating Hormone
GO	Gene Ontology
GSK3β	Glycogen Synthase Kinase 3 Beta
i-tRF	Internal tRF
ICM	Inner Cell Mass
IU	International Units
KEGG	Kyoto Encyclopedia of Genes and Genomes
LCS	let-7 complementary sites
LOS	Large Offspring Syndrome
MAPK	Mitogen-Activated Protein Kinase
miRISC	miRNA-Induced Silencing Complex
miRNA	Micro RNA
mRNA	Messenger RNA
OV	Ovoid
p53	Tumor Protein p53
PC	Principal Component
PCA	Principal Component Analysis
PGF2α	Prostaglandin F2 alpha
PI3K-Akt	Phosphoinositide 3-Kinase-Protein Kinase B (Akt) signaling pathway
RAS	Rat Sarcoma (family of small GTPases)
RNA	Ribonucleic Acid
SFRP2	Secreted Frizzled-Related Protein 2
sncRNA	Small noncoding RNAs
TE	Trophoblast
TGF-β	Transforming Growth Factor-Beta
tRF	tRNA Fragments
tRNA	Transfer RNA
TUB	Tubular
VST	Variance Stabilizing Transformation
YBX1	Y-Box Binding Protein 1
